# Supplementary material for: Causes and consequences of sex-chromosome turnovers in Diptera
Source: Evol Lett. 2026 Mar 12;10(3):253–68. doi: 10.1093/evlett/qrag003 (PMC13231274; doi:10.1093/evlett/qrag003)
Supplement: qrag003_Supplemental_Files [file qrag003_supplemental_files.zip › Supplementary_Datasets.zip!/Supplementary_Datasets/Dataset_2_Graphical_representation_outgroups/Rhagionidae_Rhagio_lineola_chromosomes.pdf]

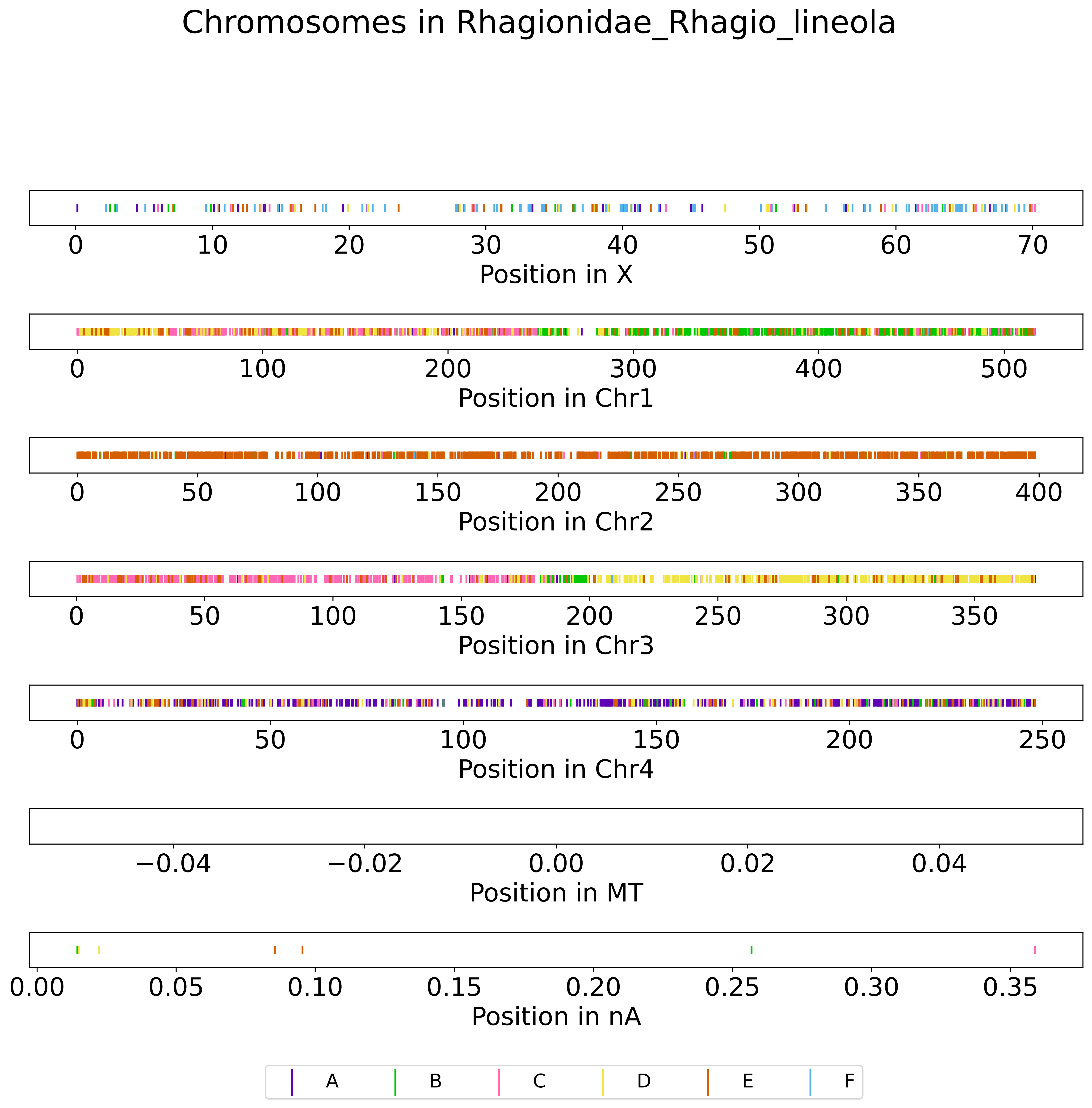
Chromosomes in Rhagionidae_Rhagio_lineola
Position in X
Position in Chr1
Position in Chr2
Position in Chr3
Position in Chr4
Position in MT
Position in nA
A
B
C
D
E
F
